# The Relation Between Cognitively Measured Executive Functions and Reported Self-Regulated Learning Strategy Use in Adult Online Distance Education

**DOI:** 10.3389/fpsyg.2021.641972

**Published:** 2021-05-04

**Authors:** Celeste Meijs, Hieronymus J. M. Gijselaers, Kate M. Xu, Paul A. Kirschner, Renate H. M. De Groot

**Affiliations:** ^1^Faculty of Educational Sciences, Open University of the Netherlands, Heerlen, Netherlands; ^2^Thomas More University of Applied Sciences, Mechelen, Belgium; ^3^Department of Complex Genetics, Faculty of Health, Medicine and Life Sciences, School for Nutrition, Toxicology and Metabolism (NUTRIM) Maastricht University, Maastricht, Netherlands

**Keywords:** executive functions, self-regulated learning strategy use, distance education, adult students, non-traditional students, age, processing speed

## Abstract

While executive functions (EFs) and self-regulated learning (SRL) strategy use have been found to be related in several populations, this relationship has not been studied in adult online distance education (ODE). This is surprising as self-regulation, and thus using such strategies, is very important here. In this setting, we studied the relation between basic executive functions (i.e., working memory and shifting, measured with cognitive tests) and reported SRL-strategy use (i.e., management of time and effort, complex and simple cognitive strategy use, contacts with others, and academic thinking) within a correlational design with 889 adult online distance students. In this study, we performed regression analyses and took age and processing speed into consideration, as processing speed and EFs decrease with age, whereas self-regulation is reported to increase with age. Cognitively measured working memory was not related to reported SRL-strategy use in adult ODE students. Thus, even though the SRL-components within the strategies seem to elicit working memory, reported SRL-strategy use is not related to the functioning of this basic EF (measured with cognitive tests). This means that if SRL-strategy use needs to be increased in adult ODE students, training of working memory might not be an effective manner for achieving that goal. Better shifting and processing speed were related to less reported SRL-strategy use, which might suggest that SRL-strategies might be used to compensate for lower shifting (in academic thinking) and lower processing speed (in simple cognitive strategy use and contacts with others). With increasing age, the number of contacts with peers or teachers decreases. This latter finding might be of relevance during the pandemic since contacts with others is importance during lockdown.

## Introduction

Self-regulated learning (SRL) is important for students enrolled in online distance education (ODE) (Artino Jr., and Stephens, 2009; Bol and Garner, 2011) because these students often study next to other responsibilities such as work, taking care of family or other persons, or community services. To be able to adhere to the study and devote enough study time to the courses, self-regulation is of utmost importance, specifically since there are less face-to-face contacts and hence less external stimulation. Moreover, executive functions (EFs), such as working memory and shifting, seem to be of importance to be able to self-regulate. The student needs to keep in mind what he or she needs to do for the study while executing other responsibilities and must be able to shift between several responsibilities. While EFs and SRL-strategy use have been found to be related in several populations (Hull et al., 2008; Hofmann et al., 2012; Roebers, 2017), this relationship has not been studied in adult ODE. Since more students enroll in ODE due to the coronavirus disease 2019 (COVID-19) pandemic lockdown (Hong et al., 2021) and SRL has been linked to academic success during the lockdown (Lestari et al., 2020), it is of even greater importance to study this relationship. Therefore, this was the main aim of the present study.

Self-regulation can be defined as a goal-directed behavior (Hofmann et al., 2012). SRL-strategies are “strategies that students use to regulate their cognition (i.e., use of various cognitive and metacognitive strategies) as well as the use of resource management strategies that students use to control their learning” (Pintrich, 1999, p. 459). Pintrich differentiates between three categories of SRL-strategies that can be used to regulate learning, namely: (1) cognitive, (2) metacognitive, and (3) resource management strategies (Pintrich et al., 1993). When using a cognitive strategy, students rehearse, organize, and elaborate on to-be-learned information. When using a metacognitive strategy, students reach a goal by planning, regulating, and monitoring their learning processes. When using a resource management strategy, students organize their environment and external resources for learning, such as time management, effort regulation, and help seeking (Pintrich et al., 1993; Kizilcec et al., 2017).

With the growing emphasis on life-long learning supported by advancements in available learning technologies, more adult students are enrolling in ODE (Rivzi et al., 2019). A characteristic of ODE is that students study at their own pace, chosen time, and place (Latanich et al., 2001; Eurydice, 2011; Eurostat, 2016) and that the study takes place via interactive telecommunication (Simonson, 2003; Broadbent and Poon, 2015). As a result, there is often less physical face-to-face contact, less guidance, and less external monitoring as compared to traditional face-to-face education. According to Bernt and Bugbee (1993, p. 100), students enrolled in ODE “often fail to monitor their progress and comprehension of course material, resulting in less-than-optimal use of limited time and effort.” Related to this, Jacobson and Harris (2008, p. 419) state that “understanding how adults learn is vital for successful adult education practice,” more specifically since ODE requires more self-regulation in students than does traditional education (Artino Jr., and Stephens, 2009; Bol and Garner, 2011). SRL-strategy use is related to academic performance in ODE (Neroni et al., 2019) and in Massive Open Online Courses (MOOCs), an often-used environment for ODE. Having SRL-skills is seen as essential for students to succeed in MOOCs (Kizilcec et al., 2017; Li, 2019).

The Motivated Strategies for Learning Questionnaire Part B (MSLQ-B), the most often used instrument for studying SRL (Panadero, 2017), was developed to study SRL-strategy use in college and high school students (Panadero, 2017; Pintrich et al., 1993). As the MSLQ-B was developed for traditional education, it was adapted for use within adult ODE (MSLQ-B DE; Meijs et al., 2019). Five categories of SRL-learning strategies were identified within adult ODE: management of time and effort, complex cognitive strategy use, simple cognitive strategy use, contacts with others, and academic thinking (see Table 1; for more information, see Meijs et al., 2019; Table A1).

**TABLE 1:** Description of the MSLQ-B DE.

MSLQ-B DE scale	MSLQ original scales	Content
Management of time and effort	• Time and study environment • Effort management	These items provide information on the amount of time spent studying and how much effort is invested.
Complex cognitive strategy use	• Rehearsal • Elaboration • Metacognitive self-regulation	The items represent handling of information, specifically based on the content, such as relating information to other stored information and monitoring of information not yet stored/understood.
Simple cognitive strategy use	• Rehearsal • Elaboration • Organization	The items represent handling of information, without the focus on the content, such as repeatedly reading the information, outlining, and summarizing.
Contacts with others	• Peer learning • Help seeking	The items refer to making contacts with peers and teachers to enhance study results.
Academic thinking	• Critical thinking • Metacognitive self-regulation • Other scales	The items regard cognitively handling of information related to their own reflection on the to-be-learned information, their own opinion, development of own ideas, and the search for alternatives.

These five SRL-learning strategies were the focus of the current study. Management of time and effort, complex cognitive strategy use, and contacts with others were found to be related with academic performance in adult ODE (Neroni et al., 2019). SRL-skills may be particularly important for adult ODE students because they do not follow a full-time, face-to-face curriculum as students in traditional education. It is thus possible that students in adult ODE may rely more on SRL.

EFs are domain general cognitive control processes that regulate behavior (Miyake and Friedman, 2012) and goal-directed activities (Cirino and Willcutt, 2017). Monitoring, goal setting, and planning are considered complex or higher-order EFs (Garner, 2009). Underlying these complex EFs, are basic EFs that facilitate complex EFs. In most studies these basic EFs are defined as updating of working memory, shifting or task switching, and inhibition (Miyake et al., 2000; Diamond, 2013). Working memory facilitates simultaneously retaining and handling information in memory (Baddeley, 2012; Hofmann et al., 2012). Shifting is the ability to switch between tasks (Hofmann et al., 2012; Diamond, 2013). Inhibition refers to the capacity to suppress reactions and impulses and prevent distraction by input from the environment (Hofmann et al., 2012; Diamond, 2013). It has been suggested that the structural functions of EF components may be subject to change over the life course. Previous studies indicate that there are only two separate basic components of EFs in older adults: working memory and shifting. Inhibition is part of a common EFs-factor (Hull et al., 2008; Miyake and Friedman, 2012; Cirino and Willcutt, 2017). Therefore, working memory and shifting were the focus of the current study, since a part of the study sample is substantially older than students in traditional educational settings. Working memory, measured as updating of working memory, has been related to academic performance in adult ODE; for shifting, this relation was not found (Gijselaers et al., 2017).

EFs have been found to be at the basis of self-regulation behavior in children and adults (Hull et al., 2008; Hofmann et al., 2012; Roebers, 2017). Higher order EFs—monitoring, goal setting, and planning (Pintrich and de Groot, 1990; Artino Jr., and Stephens, 2009; Garner, 2009; Credé and Phillips, 2011)—are needed for implementing SRL-strategies. The relation between SRL/SRL-strategy use and EFs has been studied in children (Roebers, 2017; Rutherford et al., 2018), adolescents (Effeney et al., 2013), and university students (mean age, 20 years) (Garner, 2009). Hofmann et al. (2012) state that failures in self-regulation may be caused by temporary reductions in basic EFs, and hence, that basic EFs are basal to self-regulation.

The precise relation between working memory and SRL-strategies is not clear from existing literature (Follmer and Sperling, 2016), but it is often assumed that the execution of SRL-strategies relies on working memory (Boekaerts, 1997; Winne and Nesbit, 2010). For instance, in children, working memory has been linked to the exchange of information between the procedure of a task that has to be executed and the simultaneous metacognitive monitoring and control processes that occurred during the task (Roebers, 2017) (i.e., working consistently for finishing a course) and in the suppression of irrelevant information that prevents reaching that goal (e.g., distraction from paying attention to activities irrelevant for reaching the goal; Hofmann et al., 2012). Additionally, working memory processes and switching costs are closely related (Liefooghe et al., 2008). Switching costs refer to resulting inferior performance when different tasks have to be executed in the same timeframe, and the person needs to quickly switch between different tasks. Working memory has been proposed by many learning theories as an important basis for learning (Sweller et al., 2011; Mayer, 2014). It acts as an information processor for the learner to relate newly taught information to prior knowledge and to solidify the newly formed schema into long-term memory. Similarly, working memory might facilitate the execution of both simple and complex cognitive strategies because these strategies entail simultaneous memorization, relating new information to previously learned information, and applying information to novel situations. In addition, it might also facilitate academic thinking in which new information has to be integrated with other information to create own opinions and ideas and think of alternatives. Contacts with others during studying refers to actions that a student undertakes to come in contact with fellow students or teachers. Since this strategy itself does not necessarily reflect complex thinking behaviors, it might therefore probably not rely on working memory.

Shifting has been found to be related to self-regulation in college students (Follmer and Sperling, 2016) and to self-regulative behavior in children (Roebers, 2017; Rutherford et al., 2018), but it has not been studied extensively in adults (Hofmann et al., 2012). Shifting, or task switching, might facilitate management of time and effort in adult ODE students because these students often have to switch between the means to pursue a goal (i.e., finish a course) and multiple other goals (i.e., job and family responsibilities; Hofmann et al., 2012). An additional problem is that task switching leads to inferior performance due to switch costs (Liefooghe et al., 2008). Shifting might be involved in complex and simple cognitive strategy use because shifting might be needed to switch between several steps during information processing, for instance, coupling newly learned information to already stored information or making lists of information that has to learned. In the same vein, it might facilitate academic thinking because information has to be approached from several angles, which implies switching between points of view. These three more cognitively oriented SRL-strategies (i.e., complex and simple strategy use and academic thinking) rely on working memory (see above), which in turn is related to shifting by means of task switching (Liefooghe et al., 2008). From this viewpoint, a relation between shifting and those three more cognitively based SRL-strategies might also be expected. As described earlier, contacts with others during studying refers to actions that a student undertakes to come in contact with fellow students or teachers and might therefore probably not rely on shifting either.

Thus, basic EFs have been suggested to be closely related to self-regulation and SRL in children and adults in general (Garner, 2009; Hofmann et al., 2012; Effeney et al., 2013; Cirino et al., 2017; Cirino and Willcutt, 2017; Roebers, 2017; Rutherford et al., 2018); however, studies specifically in adult ODE are lacking. Insight in these relations might be helpful to guide students who do not make use of effective SRL-strategies. EFs can be trained, at least to some extent (Hofmann et al., 2012; Diamond, 2013); however, transfer to other areas than that particular EF is questionable (Hofmann et al., 2012; Resch et al., 2019). If there is a relation between basic EFs and SRL-strategy use, then training basic EFs might lead to better self-regulation (Hofmann et al., 2012) and possibly better SRL-strategy use.

ODE students are often adults, whose ages vary from young adulthood to seniors (Neroni et al., 2015; Brand-Gruwel et al., 2016), which is a different population than the populations where a relation between EFs and SRL-strategy use is most often studied. With increasing age, students’ life experiences increase, which in turn facilitates better self-regulation, such as planning and monitoring (Justice and Dornan, 2001; Jacobson and Harris, 2008; Li, 2019). However, help seeking is reported to decrease with increasing age, at least among MOOC participants (Kizilcec et al., 2017; Li, 2019). On the other hand, cognitive capacity, such as working memory (Hull et al., 2008) and shifting (van der Elst et al., 2006a; Albinet et al., 2012), decreases with age. If this is the case, then the use of SRL-strategies that rely on these EFs might also decrease. In other words, age should be taken into account if the relation between EFs and SRL-strategy use is studied in an adult ODE population.

Information processing speed also has been found to decrease with age (Salthouse, 1996; Lezak et al., 2004; van der Elst et al., 2006b; Albinet et al., 2012; Ebaid et al., 2017). Processing speed is a basic requirement for SRL and cognitive processing (Boekaerts, 1997, 2017). More specifically, it is fundamental to the processing of cognitive tasks (Kail and Salthouse, 1994) because it determines the speed at which cognitive operations can be performed (van der Elst et al., 2006b; Albinet et al., 2012) and affects the way information is acquired and processed (Fisher et al., 2017). Higher-order cognitive tasks require sufficient cognitive processing speed (Ebaid et al., 2017). Insufficient processing speed has been found to lead to impairments in basic and higher-order complex cognitive processes, including working memory (Salthouse, 1996). Processing speed is also related to shifting, since temporal constraints determine the quality of performance if processes that have to be executed sequentially can be executed without time pressure (Liefooghe et al., 2008). However, a lower processing speed leads to time pressure and might thus lead to lower performances. These relations between processing speed and both working memory and shifting might, in turn, lead to less use of complex and simple cognitive strategies such as elaboration, abstraction, and integration, and academic thinking. In other words, processing speed might also be related to both EFs and SRL-strategy use and should be considered if the relation between EFs and SRL-strategy use is studied in an adult ODE population.

Enrollment in ODE for adult populations is increasing. Most studies on the relation between basic EFs and SRL have been carried out in children and college students (see above). The aim of the current study was to investigate the relation between basic EFs (measured with cognitive tests) and self-reported SRL-strategy use in adult ODE, with consideration of age and processing speed, within an observational study design. We hypothesized that:

1. Better basic EFs (i.e., working memory and shifting measured with cognitive tests) would predict more reported use of management of time and effort, complex and simple cognitive strategies, and academic thinking.
2. Basic EFs (i.e., working memory and shifting measured with cognitive tests) would not predict reported contacts with others.

## Materials and Methods

### Procedure

The current observational study was part of the Adult Learning Open University Determinants (ALOUD) study (Neroni et al., 2015). The aim of ALOUD was the investigation of the biological and psychological determinants of learning in adult ODE students. Students who registered in the period of August 2012 to August 2013 (*N* = 4,945) at the Open University in the Netherlands (OUNL) were approached to participate in the study. ALOUD makes use of a baseline measure including a questionnaire and cognitive tests and a follow-up measurement at 14 months using only a questionnaire. Of the students approached for participation, 57.5% responded (*n* = 2,842), 41.3% completely filled out the questionnaire and the cognitive tests at baseline (*n* = 2,040), and 1,196 students (24.2%) also participated in the follow-up measurement (for more information regarding ALOUD and the procedure, see Neroni et al., 2015). The characteristics of the responding participants at baseline were comparable to the general population of students who study at OUNL (Moerkerke, 2014) with respect to, for instance, employment and living circumstances (i.e., single, with a partner, with or without children). ALOUD was approved by the Ethics Committee of OUNL.

### Participants

Students who completed the entire questionnaire at baseline as well as the MSLQ-B DE on the second measurement (*N* = 1,196) were included. Students who remarked at the end of the questionnaire at the second measurement that they had not been active students during the 14-month timeframe (*n* = 42) were excluded. Students who had not completed the cognitive measures or had scores that suspected unreliable data (see *Cognitive Tests*) or who made remarks that indicated unreliable data were excluded from the dataset (*n* = 265). This resulted in a sample of 889 students (mean age = 38.91; range, 19-69 years; *SD* = 11.42; 555 female (62.4%), 334 male).

### Courses

The study was executed among all courses of the OUNL. A characteristic of these courses is that they are online, distance education courses that can be studied individually on the student’s own pace, time, and location. Contacts with teachers and fellow students can be initiated by the students.

### Materials

#### SRL-Strategy Use

The MSLQ-B DE was used to inquire SRL-strategy use. It consists of five subscales: management of time and effort, complex cognitive strategy use, simple cognitive strategy use, contacts with others, and academic thinking (see Table A1). The MSLQ-B DE has a relatively good reliability in terms of internal consistency (α’s of the subscales ranging from 0.70 to 0.80) (Meijs et al., 2019). The items were scored on a 7-point Likert scale, ranging from 1 (totally disagree) to 7 (totally agree). MSLQ-B DE data gathered at the follow-up measurement were considered more valid than data collected at baseline. The rationale was that when students have acquired study experience at the follow-up measurement and can base their strategy report on the obtained grade, they can better assess their learning strategy use (Credé and Phillips, 2011) than when they just started their study at baseline measurement. Mean scores (after reversal of the reversed items) of the five subscales were used in the current study as measures of SRL-strategy use.

#### Cognitive Tests

Three cognitive tests were administered online at the end of the questionnaire at baseline. In advance, the students were instructed to use an attached mouse; thus, students using trackballs, track points, touch pads, or other tracking devices could not participate. This requirement was made to minimize device-dependent variations. These specific cognitive tests were chosen because they are well known and often used paradigms, which could be administered in an ODE setting with valid measurements.

##### Working Memory

Working memory was in the current study conceptualized by updating. It was measured with a computerized N-Back Test (NBT; Lezak et al., 2004), which was developed by Dobbs and Rule (1989). The NBT specifically measures the focal attention in working memory, namely, the maintenance of previous items, while simultaneously attending to the current item (van Gerven et al., 2007). The test includes high task demands in that there is a simultaneous maintenance and updating of task-relevant information (Hofmann et al., 2012). In the paradigm used in the current study, the student was presented a series of digits and had to indicate, as fast and accurate as possible, whether the current presented digit was the same as the digit that was presented two positions back. In total, the tests consisted of 60 items. During the test, the students placed their index fingers on the “A”-key (left for “Yes”) and on the “L”-key (right for “No”) of their keyboard and had to press the corresponding letter to indicate whether the digit was the same or not. Reminder of “A = Yes” and “L = No” remained visible during the test in, respectively, the left and right corner of the screen. The first two items had to be answered with “No” because there were no previous items to compare to. The number of correctly identified items in the test session was used as a measure for working memory, with a higher score representing a better working memory. Students that scored below chance level (i.e., 30 items correct) were not included in the study because scoring below chance level indicates unreliable test data.

##### Shifting

Shifting was measured with the Trail Making Test (TMT) that was originally designed for the Army Individual Test Battery (1944). The reliability coefficients vary, but in most reports, the reliability coefficient was approximately 0.80 (Lezak et al., 2004). The TMT consists of two parts: “A” and “B.” In the current study, in part “A,” 25 dots with the numbers 1–25 were placed throughout the screen in an asymmetrical, random order. The student had to click with the mouse on the dots in increasing order, as fast as possible. In part “B,” dots with numbers and letters were placed in a similar, asymmetrical, random order. The participant had to click on the dots, starting with a number and then a letter, then again a number, then a letter in increasing order and alphabetical order, respectively (e.g., 1, A, 2, B, 3, C, etc.). Before the actual test session, there were training sessions (consisting of seven items per part) and the student received feedback after each session. The “B–A” score has been reported validly to be strongest related to switching capacity (Sánchez-Cubillo et al., 2009) and to reflect the costs of switching between concepts (van der Elst et al., 2006a). Therefore, in the current study, the time needed for part “B”–part “A” (in seconds) was used as a measure for shifting, with a lower score representing better shifting. Students with a negative score (i.e., time needed for part “A” was longer than the time needed for part “B”) were not included in the study because a negative score indicates unreliable test data.

##### Processing Speed

Processing speed was measured with the Symbol Digit Substitution Test (SDST), which is an adapted version of the Letter Digit Substitution Test (LDST; van der Elst et al., 2006b) and the Symbol Digit Modalities Test (SDMT; Smith, 1991; Lezak et al., 2004). The LDST and SMDT have good test–retest reliabilities (Lezak et al., 2004; van der Elst et al., 2006b). In the SDST, the paradigm that was used in the current study, a symbol had to be matched to a number based on a displayed key. This key appeared at the top of the screen. Below the key, there was a 3 × 3 matrix, containing the numbers 1–9. After clicking on a number within the matrix, a (next) symbol from the key appeared, and the students had to click on the matching number within the matrix, as fast as possible, for 90 s. The paradigm measures were, among others, perceptual processing (Sánchez-Cubillo et al., 2009), cognitive processing speed, speed of mental operation, and psychomotor speed (Ebaid et al., 2017). Before the actual test session, there was a practice session. In the current study, the number of correct matches was used as a measure of processing speed, with a higher score representing a faster processing speed.

### Statistics and Analyses

#### Extreme Values and Missing Data

First, we explored the SRL-strategy use data for missing data and extreme values. Extreme values were defined as minimally three times the interquartile distance above the 75th percentile or below the 25th percentile (Huizingh, 2002).

#### Analyses

All analyses were conducted using SPSS 24.0.0 (Chicago, IL, United States). The assumptions for conducting multiple regression analyses (MRAs) were checked. Five MRAs were performed to study whether basic EFs, measured with cognitive tests at baseline, predicted reported SRL-strategy use, measured at follow-up in adult ODE students, and what the roles of age and processing speed were in the prediction of SRL-strategy use. Mean scores on the SRL-strategy use scales (i.e., management of time and effort, simple and complex cognitive strategy use, contacts with others, and academic thinking) were the outcome measures. Age (in years) and processing speed were entered in the first block (model 1), and the EFs were added in the second block (model 2). βs of ≤ 0.1 were classified as negligible, between 0.1 and 0.3 as small/weak, between 0.3 and 0.5 as medium, and ≥ 0.5 as strong/high (Cohen, 1992; Field, 2009). Only βs above 0.1 are interpreted. All tests were performed with an α-level of 0.05, two-sided testing.

## Results

Missing data were not present in the dataset due to the sampling method (see *Participants*). Extreme values were found for complex cognitive strategy use (*n* = 4), processing speed (*n* = 2), working memory (*n* = 34), and shifting (*n* = 12). These extreme values were replaced with the highest/lowest value within the range. The assumptions for MRA were met, except for complex cognitive strategy use, where there might be some violation of independence and normal distribution of errors.

Descriptives of the variables are displayed in Table 2. As can be seen, contacts with others is the least reported and complex cognitive strategy use the most. For working memory, no values below 30 were reported, and for shifting, no negative values were reported, indicating that the suspected unreliable data were removed from the dataset.

**TABLE 2:** Descriptives of the variables.

Variable	Mean	*SD*	Range
Management of time and effort	4.868	1.115	1.33–7
Complex cognitive strategy use	5.207	0.794	1.80–7
Simple cognitive strategy use	4.940	1.169	1–7
Contacts with others	2.581	1.208	1–7
Academic thinking	4.171	1.090	1–7
Working memory	55.875	4.739	42–60
Shifting	20.055	12.379	0.05–65.37
Processing speed	49.494	8.350	16–86
Age	38.911	11.420	19–81

**FIGURE 1:**
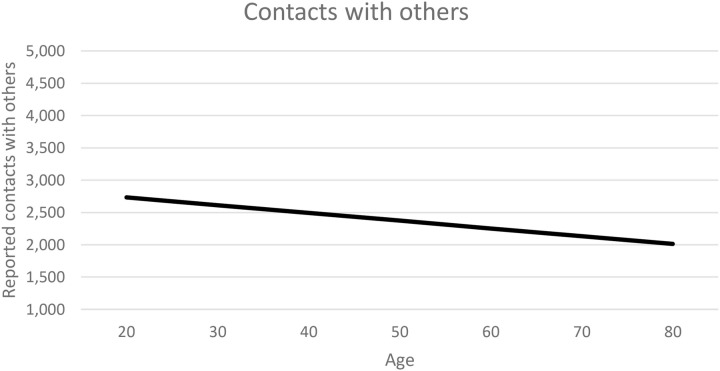
Mean reported contacts with others estimated by the regression model for students with an average processing speed.

The regression statistics are displayed in Table 3. Management of time and effort and complex cognitive strategy use were not significantly predicted by the factors in the models, which indicates that there was no significant relation between the cognitive measures and age and reported use of these two SRL-strategies. Simple cognitive strategy use and contacts with others were significantly predicted by model 1 (age and processing speed), which indicates that there was a relation between age and/or processing and reported use of these two SRL-strategies. For both strategies, model 2 (age, processing speed, and the EFs) was significant also but did not significantly predict better than model 1. An increase in processing speed weakly predicted a decrease in simple cognitive strategy use and contacts with others, and an increase in age weakly predicted a decrease in contacts with others (see Figure 1). Academic thinking was significantly predicted by model 2 (age, processing speed, and the EFs) by shifting. This indicated that a higher shifting weakly predicted less academic thinking.

**TABLE 3:** Statistics for the multiple regression analyses (MRAs).

	Management of time and effort				Complex cognitive strategy use				Simple cognitive strategy use				Contacts with others				Academic thinking			
	*B*	*SE*	*β*	*p*	*B*	*SE*	*β*	*p*	*B*	*SE*	*β*	*p*	*B*	*SE*	*β*	*p*	*B*	*SE*	*β*	*p*
**Model 1**																				
Constant	5.080	0.392		0.000***	5.249	0.286		0.000***	6.065	0.409		0.000***	3.835	0.424		0.000***	3.661	0.384		0.000***
Age	0.003	0.004	0.031	0.456	0.002	0.003	0.022	0.605	−0.004	0.004	−0.042	0.314	−0.012	0.004	−0.114	0.006**	0.007	0.004	0.074	0.075
Processing speed	−0.007	0.006	−0.050	0.231	−0.002	0.004	−0.022	0.601	−0.019	0.006	−0.138	0.001**	−0.016	0.006	−0.110	0.008**	0.005	0.005	0.036	0.383
	*Model: F*(2,886) = 2.356,				*Model: F*(2,886) = 0.666,				*Model: F*(2,886) = 6.303,				*Model: F*(2,886) = 4.547,				*Model: F*(2,886) = 1,610			
	*p* = 0.095, *R*^2^ = 0.005				*p* = 0.514, *R*^2^ = 0.002				*p* = 0.002**, *R*^2^ = 0.014				*p* = 0.011*, *R*^2^ = 0.010				*p* = 0.200, *R*^2^ = 0.004			
**Model 2**																				
Constant	4.700	0.601		0.000***	5.001	0.438		0.000***	7.056	0.627		0.000***	4.251	0.649		0.000***	3.451	0.584		0.000***
Age	0.003	0.004	0.029	0.483	0.001	0.003	0.017	0.683	−0.004	0.004	−0.036	0.383	−0.012	0.004	−0.115	0.006**	0.006	0.004	0.064	0.123
Processing speed	−0.007	0.006	−0.055	0.219	−0.001	0.004	−0.009	0.835	−0.019	0.006	−0.134	0.003**	−0.012	0.006	−0.086	0.053*	0.011	0.006	0.085	0.056
Working memory	0.007	0.009	0.030	0.423	0.002	0.006	0.014	0.710	−0.017	0.009	−0.068	0.064	-0.011	0.009	−0.044	0.232	−0.005	0.008	−0.022	0.556
Shifting	0.001	0.003	0.016	0.666	0.003	0.002	0.052	0.130	−0.005	0.004	−0.057	0.132	0.002	0.004	0.025	0.511	0.011	0.003	0.121	0.001**
	*Model: F*(4,884) = 1.350,				*Model: F*(4,884) = 0.802,				*Model: F*(4,884) = 4.303,				*Model: F*(4,884) = 2.883,				*Model: F*(4,884) = 3.970,			
	*p* = 0.249, *R*^2^ = 0.006,				*p* = 0.524, *R*^2^ = 0.004,				*p* = 0.002**, *R*^2^ = 0.019,				*p* = 0.022*, *R*^2^ = 0.013,				*p* = 0.003**, *R*^2^ = 0.018,			
	*F Change*(2,884*)* = 0.348,				*F Change*(2,884*)* = 0.938,				*F Change*(2,884) = 2.285,				*F Change*(2,884) = 1.218,				*F Change*(2,884) = 6.3117,			
	*p* = 0.706				*p* = 0.392				*p* = 0.102				*p* = 0.296				*p* = 0.002**			

*^∗^p < 0.05, ^∗∗^p < 0.01, ^∗∗∗^p < 0.001.*

In sum, shifting was related to academic thinking. The other EFs were not related to reported SRL-strategy use. Simple cognitive strategy use was related to processing speed. Contacts with others was related to age and processing speed. Management of time and effort, complex cognitive strategy use, and academic thinking were not related to age and processing speed.

## Discussion

SRL is important for students enrolled in ODE (Artino Jr., and Stephens, 2009; Bol and Garner, 2011) because these students often study next to other responsibilities such as work, taking care of family or other persons, or community services. To be able to adhere to the study and devote enough study time to the courses, self-regulation is of utmost importance, specifically since there are less face-to-face contacts and hence less external stimulation. The courses in ODE have to be studied individually, in the time, pace, and location chosen by the student, which requires a large amount of SRL. Moreover, EFs, such as working memory and shifting, seem of importance to self-regulate. The student needs to keep in mind what he or she needs to do for the study while executing the other responsibilities and must be able to shift between the several responsibilities. While EFs and SRL-strategies use have been found to be related in several populations (Hull et al., 2008; Hofmann et al., 2012; Roebers, 2017), this relationship has not been studied in adult ODE. Therefore, the aim of the current study was to investigate the relation between basic EFs (measured with cognitive tests) and reported SRL-learning strategy use within adult ODE, with consideration of age and processing speed. Overall, basic EFs were not related to reported SRL-strategy use. However, higher shifting was related to less academic thinking. Additionally, with an increase in processing speed, there was a decrease in reported simple cognitive strategy use and contacts with others. With increasing age, there was a decrease in reported contacts with others.

### Basic EFs and the Relation With SRL-Strategy Use

Students with a better shifting capacity reported less academic thinking. This is against the expectations since it has been reported that better shifting facilitated academic thinking. During academic thinking, information has to be considered from several viewpoints, which implies shifting. That students with a lower capacity reported more academic thinking might be caused by the time needed for considering information from various viewpoints. If a student is able to shift quickly, perhaps less time is needed for academic thinking, leading to lower reports. Further studies are needed to clarify this finding. Besides the contradictory finding regarding shifting and academic thinking, no relations between EFs and SRL-strategy use were found. Thus, overall, this suggests that there is no relation between basic EFs (measured with cognitive tests) and reported SRL-strategy use in adult ODE students. Working memory was assumed to be involved in SRL-strategy use (Boekaerts, 1997; Winne and Nesbit, 2010); more specifically, Hofmann et al. (2012) found that working memory is involved in the top–down control of attention to pursue a goal and in the suppression of irrelevant information that prevents reaching that goal and was therefore expected to facilitate time and effort management. In addition, simultaneous storage and manipulation of information takes place in working memory (Baddeley, 2012), actions necessary to execute complex and simple cognitive strategy use and academic thinking. These more cognitively based strategies involve, among others, memorization, keeping track of stored information, relating new information to previously stored information, reflection on the to-be-learned information with regard to their own opinion, development of own ideas, and the search for alternatives.

Shifting is needed to switch between multiple goals (Hofmann et al., 2012), such as finishing a course, managing the household, and working, and was therefore expected to facilitate management of time and effort. Shifting was also assumed to be involved in simple and complex cognitive strategies because it might be needed to switch between the mental operations needed to execute the strategies.

A possible explanation for lack of a relation between EFs and SRL-strategy use might be that working memory and shifting were assessed by means of cognitive tests (N-back test and TMT, respectively), whereas SRL-strategy use was measured by means of self-report (MSLQ-B DE). It supports previous studies into the convergent validity between several types of measures of self-control that showed that the correlation between self-reports and cognitive tests is low (Duckworth and Kern, 2011). Thus, if the basic EFs would had been measured via self-report of behavior that relies on the functioning of basic EFs, perhaps relations between basic EFs and reported SRL-strategy use would have been found. Another explanation might be that basic EFs are indeed involved in the use of these SRL-strategies but that other factors are far more important for SRL-strategy use in adult ODE than basic EFs. For instance, many, if not most, adult students following ODE have jobs and other responsibilities, making studying probably not their top priority (Eurydice, 2011; Eurostat, 2016). In other words, studying might be carried out under time pressure, which makes the conditions of studying for adult ODE students different from college students and children.

Additionally, the current study confirmed that basic EFs (i.e., working memory and shifting measured with cognitive tests) were not related to reported contacts with others. The items in the scale “contacts with others” refer to working together, discussing the materials, identifying students that might be able to help, and asking for clarification from teachers (see Table 1 and Table A1). These activities do not necessarily elicit basic EFs.

### Age and Processing Speed and the Relation With SRL-Strategy Use

Kizilcec et al. (2017) and Li (2019) reported decreased help seeking with increasing age in MOOCs. This was confirmed here for adult ODE students in which with increasing age, students reported to make less use of contacts with others (see Figure 1). There was a difference of 0.72 (on 7-point scale) between students aged 20 and students aged 80. Perhaps, older students might have other study motives and more intrinsic motivation to study, whereas younger students might have more extrinsic motives (i.e., grades and career opportunities) and need to succeed to obtain their goal. Therefore, older students might not be as interested in contacts with peers and teachers as younger students.

No relations were found between age and the other reported SRL-strategies. On the one hand, SRL-strategies that rely on the use of basic EFs might decrease based on a decrease in basic EFs with age (van der Elst et al., 2006a; Hull et al., 2008; Albinet et al., 2012). This was not confirmed here and is in line with the finding that there is no relation between basic EFs (measured with cognitive tests) and reported SRL-strategy use. On the other hand, SRL-strategy use might increase with age because of more life experiences that would lead to better SRL (Justice and Dornan, 2001; Jacobson and Harris, 2008; Li, 2019). This was also not confirmed here. Thus, even though SRL is suggested to increase with age, this is not related to reported SRL-strategy use in adult ODE students.

Processing speed is fundamental for cognitive processing (Kail and Salthouse, 1994; van der Elst et al., 2006b; Albinet et al., 2012) and could therefore be related to the use of SRL-strategies with cognitive components such as complex and simple cognitive strategy use and academic thinking. However, in the current study, we found that with increasing processing speed, students reported to make less use of simple cognitive strategies and to have fewer contacts with others. Thus, the assumption that processing speed is fundamental for cognitive SRL-strategies was not confirmed for adult ODE students. Moreover, the relation that was found, namely that with increasing processing speed, reported simple cognitive strategy decreased, was in the opposite direction than expected. Perhaps, as with shifting and academic thinking, a faster processing speed results in less time needed to execute simple cognitive strategies. The SRL-strategies might perhaps be used to compensate for lower cognitive functions. More studies are needed to explain why this was only found for simple cognitive strategy use and not for the other SRL-strategies with cognitive components. Finally, increased processing speed was related to decreased reported contacts with others. This was unexpected because contacts with others do not contain a cognitive factor. This also requires more investigation.

## Limitations and Future Studies

Some issues within the current study need special attention. First, the cognitive tests were administered online with no supervision of researches and hence no control. To minimize the occurrence of errors, students were instructed about the correct equipment needed to execute the tests (see *Cognitive Tests*), and the data were carefully explored for possible unreliable data (see *Participants* and *Cognitive Tests*). Second, even though inhibition is not a separate basic EF in older adults (Hull et al., 2008; Miyake and Friedman, 2012; Cirino and Willcutt, 2017), inhibition should be included in future studies in adult DE because this also includes younger adults, which would give more insight into the relations between basic EFs (measured with cognitive tests) and reported SRL-strategy use. Third, in the current study, we included self-reported SRL-strategy use. In future studies, objective measures of SRL-strategy use, such as learning analytics data or learning management systems (Maldonado-Mahauad et al., 2018; Rodrigues et al., 2019), should be included to obtain more objective measures of SRL-strategy use instead of possibly biased self-reports. Additionally, self-report measures of EFs could be included.

## Conclusion

Cognitively measured working memory was not related to reported SRL-strategy use in adult ODE students. Thus, even though the SRL-components within the strategies seem to elicit working memory, reported SRL-strategy use is not related to the functioning of this basic EF (measured with cognitive tests). This means that if SRL-strategy use needs to be increased in adult ODE students, training of working memory might not be an effective manner for achieving that goal. Better shifting and processing speed were related to less reported SRL-strategy use, which might suggest that SRL-strategies might be used to compensate for lower shifting (in academic thinking) and lower processing speed (in simple cognitive strategy use and contacts with others). With increasing age, the number of contacts with peers or teachers decreases. This latter finding might be of relevance during the pandemic since contacts with others is importance during lockdown.

## Data Availability Statement

The dataset presented in this article is not readily available because the data used for the current article is part of a larger dataset that is securely stored on an institutional server. It is thus not possible for the data to be publicly available from open servers free to download. Requests to access the datasets should be directed to RDG, renate.degroot@ou.nl.

## Ethics Statement

The study involving human participants was reviewed and approved by cETO, Ethics Committee of the Open University. The participants provided their written informed consent to participate in this study.

## Author Contributions

CM designed and performed the analysis and wrote the manuscript. RDG designed ALOUD. HG performed the data collection of ALOUD. KX, HG, PK, and RDG provided critical input on the design and substantially improved the manuscript. All authors contributed to the article and approved the submitted version.

## Conflict of Interest

The authors declare that the research was conducted in the absence of any commercial or financial relationships that could be construed as a potential conflict of interest.

## Appendix

**TABLE A1:** Items of the MSLQ-B DE ordered according to SRL-strategies.

**Management of time and effort**
I make good use of my study time for this course.
When course work is difficult, I either give up or only study the easy parts. (REVERSED)
I make sure that I keep up with the weekly readings and assignments for this course.
I attend this course regularly.
Even when course materials are dull and uninteresting, I manage to keep working until I finish.
I rarely find time to review my notes or readings before an exam. (REVERSED)
**Complex cognitive strategy use**
I memorize key words to remind me of important concepts in this course.
I try to think through a topic and decide what I am supposed to learn from it rather than just reading it over when studying for this course.
When reading for this course, I try to relate the material to what I already know.
When studying for this course I try to determine which concepts I don’t understand well.
I try to apply ideas from course readings in other course activities such as lectures and discussion.
**Simple cognitive strategy use**
When I study the readings for this course, I outline the material to help me organize my thoughts.
When studying for this course, I read my course notes and the course readings over and over again.
When I study for this course, I go over my course notes and make an outline of important concepts.
When I study for this course, I write brief summaries of the main ideas from the readings and my course notes.
I make lists of important items for this course and memorize the lists.
**Contacts with others**
I try to work with other students from this course to complete the course assignments.
When studying for this course, I often set aside time to discuss course material with a group of students from the course.
I ask the instructor to clarify concepts I don’t understand well.
I try to identify students in this course whom I can ask for help if necessary.
**Academic thinking**
When reading for this course, I make up questions to help focus my reading.
I often find myself questioning things I hear or read in this course to decide if I find them convincing.
When a theory, interpretation, or conclusion is presented in this course or in the readings, I try to decide if there is good supporting evidence.
I treat the course material as a starting point and try to develop my own ideas about it.
Whenever I read or hear an assertion or conclusion in this course, I think about possible alternatives.
